# The influence of filtering and downsampling on the estimation of transfer entropy

**DOI:** 10.1371/journal.pone.0188210

**Published:** 2017-11-17

**Authors:** Immo Weber, Esther Florin, Michael von Papen, Lars Timmermann

**Affiliations:** 1 Department of Neurology, University Hospital Giessen & Marburg, Marburg, Germany; 2 Institute of Clinical Neuroscience and Medical Psychology, Medical Faculty, Heinrich-Heine-University, Düsseldorf, Germany; 3 Institute of Geophysics & Meteorology, University of Cologne, Cologne, Germany; Lanzhou University of Technology, CHINA

## Abstract

Transfer entropy (TE) provides a generalized and model-free framework to study Wiener-Granger causality between brain regions. Because of its nonparametric character, TE can infer directed information flow also from nonlinear systems. Despite its increasing number of applications in neuroscience, not much is known regarding the influence of common electrophysiological preprocessing on its estimation. We test the influence of filtering and downsampling on a recently proposed nearest neighborhood based TE estimator. Different filter settings and downsampling factors were tested in a simulation framework using a model with a linear coupling function and two nonlinear models with sigmoid and logistic coupling functions. For nonlinear coupling and progressively lower low-pass filter cut-off frequencies up to 72% false negative direct connections and up to 26% false positive connections were identified. In contrast, for the linear model, a monotonic increase was only observed for missed indirect connections (up to 86%). High-pass filtering (1 Hz, 2 Hz) had no impact on TE estimation. After low-pass filtering interaction delays were significantly underestimated. Downsampling the data by a factor greater than the assumed interaction delay erased most of the transmitted information and thus led to a very high percentage (67–100%) of false negative direct connections. Low-pass filtering increases the number of missed connections depending on the filters cut-off frequency. Downsampling should only be done if the sampling factor is smaller than the smallest assumed interaction delay of the analyzed network.

## Introduction

Understanding the connectivity and directional interaction of different brain areas is highly relevant in order to gain further insight into brain function. In electrophysiological research Granger causality [1] and its multivariate extensions such as partial directed coherence [2], have been applied for this aim, resulting in extensive progress in understanding information flow in the healthy [3–6] and pathological brain alike [7–11]. One disadvantage of classical Granger causality is the need for a linear autoregressive model. Therefore, Granger causality cannot always properly identify nonlinear interactions. While Wold’s theorem states that even nonlinear covariance stationary time-series can be represented as an infinite order moving average process [12], this MA process might lead to an infinite order VAR process in the observables. Only if this infinite order VAR can be sufficiently approximated by a finite order VAR, i.e. the influence of past time points quickly decays, then classic Granger causality can correctly detect causal influences. However, as one does not know a priori whether a finite order VAR suffices, classic Granger causality might not be the optimal choice for detecting nonlinear interactions.

Due to its high complexity, the brain is believed to be nonlinear on many spatial and temporal scales. One prominent example of a nonlinear phenomenon in the human brain is synchronization of recorded electrophysiological neuronal activity [13–16]. Synchronization requires, that the participating systems are self-sustained oscillators, which are described by nonlinear differential equations [15]. Other nonlinear phenomena include stochastic resonance [17], multistability [18] and even chaos [19]. Although linear relationships are generally more robustly detected they are probably only a small part of the rich dynamics of the human brain. Using nonlinear analysis tools may thus prove to be insightful in order to understand the brain’s more complex behavior, especially since advances in computational capabilities over the last decade made them more applicable. In order to deal with such nonlinearities in electrophysiological data (for general reviews see [20,21]), the measure of transfer entropy (TE) has been developed [22]. Since its introduction, TE has been widely applied in neuroscientific modeling studies, animal studies and human studies[23–31].

For many neuroscientific research purposes it is common to preprocess the acquired data. Although TE is being continuously developed and used as a tool for neuroscientific research [32–35], little is known about the influences of filtering and downsampling on its estimation. This, however, is especially important for the analysis using TE. In contrast to Granger causality, which has also spectral representations, so far, no spectral decomposition is possible using TE. Thus the, so far, only way to limit estimation of information transfer using TE on a specific frequency band is to filter the data prior to the analysis. For Granger based methods filtering and downsampling have a tremendous effect on falsely detected connections [36]. Barnett and Seth [37] explained the increase of false negatives and false positives after filtering by an increase of the autoregressive model order. While filtering might have a strong influence on estimation of Granger causality, the population statistics are invariant if a causal invariant filter is used [37]. In contrast, downsampling even distorts the population statistics [38]. Since Barnett et al. [39] proved that TE is a generalization of Granger causality, with both being completely equivalent for jointly Gaussian distributed processes, one might expect a similar negative influence of preprocessing on TE estimation as for Granger causality. However, the estimators for Granger based methods and TE are highly different, with the former usually being a parametric approach where it is necessary to optimize model fitting [1] and the latter estimating probability distributions [22]. Only recently nonparametric Granger causality measures have been introduced [40]. Though one has to keep in mind that, while these measures are nonparametric in the sense that they are not estimated from an autoregressive model, but from the cross-power spectrum, they are still linear measures as the cross-power spectrum is a second order statistic. Also, new state-space methods for Granger causality have been recently introduced, which offer smaller estimation biases and higher statistical power in comparison to standard autoregressive modelling [41,42]. In conclusion, preprocessing might have different effects on estimating predictive information transfer in (parametric) Granger causality and TE.

For the purpose of this study, a range of preprocessing parameters including different filter types, filter settings and downsampling factors were tested. As TE is capable of detecting not only linear but also nonlinear dependencies, we tested TE with a modified version of the linear Kus-model [43] as well as two nonlinear models with a logistic [44] and a sigmoid coupling function [45]. We hypothesize that filtering leads to an increased degree of detected spurious information flow dependent on the filter properties, i.e. the cut-off frequency and order, and the analyzed system’s dynamics, i.e. the degree of nonlinearity. Additionally, we speculate that filtering has a negative influence on the estimation of the interaction delay dependent on the filter order. This simulation study aims to provide guidelines on how to preprocess electrophysiological data in order to reliably estimate transfer entropy.

## Methods

### Ethics statement

The patient of whom data was used in this study gave written informed consent to the recording of EEG data. The data collection was approved by the local ethics committee of the medical faculty in cologne (study no. 14–129) and conducted in accordance with the Declaration of Helsinki.

### Transfer entropy

For the remainder of this paper, let us define {x_1_,…,x_T_} and {y_1_,…,y_T_} as finitely sampled time series generated by measurements of coupled neurobiological systems, e.g. electrical activity of brain areas. These time series are understood as realizations x_t_, y_t_ of discrete random variables X_t_, Y_t_ at discrete times t = 1…T. The random variables generate random processes *X*, *Y*. Normal case letters indicate scalar values while bold letters refer to their vector valued state space representations. A state is a vector that collects all past realizations of a current observable necessary for prediction. The d_x_-dimensional state vector $x_{t}^{d_{x}}$ at time t is defined as:

$$x_{t}^{d_{x}}=\left[{\text{x}}_{\text{t}-\left(d_{x}-1\right)\tau },{\text{x}}_{\text{t}-\left(d_{x}-2\right)\tau },\ldots \;,{\text{x}}_{\text{t}-\tau },{\text{x}}_{\text{t}}\right],$$ (1)

with time delay τ.

The average information content of a continuous random variable X can be defined as the Shannon entropy H(X) according to:

$$H\left(\text{X}\right)=-\int p\left(\text{x}\right){\text{log}}_{a}p\left(\text{x}\right)dx,$$ (2)

with p(x) the probability density function of the random variable X. The base is usually chosen to be a = 2 in order to interpret the information content in bits. While for discrete variables, H(X) is always positive semidefinite, H(X) may be negative for continuous variables. Based on the Shannon entropy the mutual information I of two variables X and Y is defined as the shared information content between both variables according to:

I(X;Y)=H(X)+H(Y)−H(X,Y), (3)

Where

$$H\left(\text{X},\text{Y}\right)=-\iint p\left(x,y\right){\text{log}}_{a}p\left(x,y\right)dx\;dy,$$ (4)

is the joint entropy and p(x,y) is the joint probability distribution of X and Y [46].

Wiener [47] defined a process *X* being causal to a process *Y* if *Y* is better predicted by incorporating past information of *X* than by using only past information of *Y*. TE is a straightforward information theoretic approach on Wiener’s principle of causality. It can be defined as the mutual information of the past state of the source process (***X***^-^) and the present of the target process (*Y*) conditioned on the past state of the target process (***Y***^-^):

$$TE\left(X\to Y\right)=I\left(X^{-};Y|Y^{-}\right),$$ (5)

where

$$I\left(X^{-};Y|Y^{-}\right)=H\left(X^{-}|Y^{-}\right)-H(X^{-}|Y,Y^{-})$$ (6)

and

$$H\left(X^{-}|Y^{-}\right)=H\left(X^{-}\right)-I\left(X^{-};Y^{-}\right).$$ (7)

The transfer entropy estimator used in this study is the one implemented in the MATLAB toolbox TRENTOOL [48]:

$$TE_{SPO}\left(X\to Y\right)=\int_{\text{yt}}\int_{y_{\text{t-1}}}\int_{x_{\text{t-u}}}p\left({\text{y}}_{\text{t}},y_{t-1}^{d_{y}},x_{t-u}^{d_{x}}\right)\;{\text{log}}_{2}\frac{p\left({\text{y}}_{\text{t}}|y_{t-1}^{d_{y}},x_{t-u}^{d_{x}}\right)}{p\left({\text{y}}_{\text{t}}|y_{t-1}^{d_{y}}\right)}dy_{t}dy_{t-1}dx_{t-u},$$ (8)

with the conditional probability $p\left(y_{t}|y_{t-1}^{d_{y}},x_{t-u}^{d_{x}}\right)$ defined as

$$p\left({\text{y}}_{\text{t}}|y_{t-1}^{d_{y}},x_{t-u}^{d_{x}}\right)=\frac{p\left({\text{y}}_{\text{t}},(y_{t-1}^{d_{y}},x_{t-u}^{d_{x}})\right)}{p\left(y_{t-1}^{d_{y}},x_{t-u}^{d_{x}}\right)},$$ (9)

where u is the delay of information transfer and d_x_ and d_y_ are the dimensions of **x** and **y**, respectively. The subscript SPO indicates that self-prediction of the target time series is optimized, i.e. that **y**_t-1_ is most predictive of y_t_. Eq (8) can be rewritten as a sum of four Shannon entropies:

$$TE\left(X\to Y\right)=H\left(y_{t-1}^{d_{y}},x_{t-u}^{d_{x}}\right)-H\left({\text{y}}_{\text{t}},y_{t-1}^{d_{y}},x_{t-u}^{d_{x}}\right)+H\left({\text{y}}_{\text{t}},y_{t-1}^{d_{y}}\right)-H\left(y_{t-1}^{d_{y}}\right).$$ (10)

Note that this estimator is bivariate, i.e. it analyzes pairs of time series. This may lead to spurious detections of information transfer due to multivariate effects like common drive or cascade effects. However, if the delay of information transfer is known, multivariate effects can be flagged by applying a graph theoretical approach [49]. As TRENTOOL is designed for trial based data all analyses in this study were done using simulated trials, i.e. multiple short independent realizations of stochastic processes, rather than a single realization of long duration.

According to Wibral et al. [50] interaction delays can be reconstructed by estimating TE over a range of possible interaction delays *u*. It has been mathematically proven that TE(*X→Y*) is maximal for two discrete-time random processes *X* and *Y* coupled from *X* to *Y* with a non-zero delay δ when *u* is equal to δ [50]. For our study a range of u = 1–100 lags was analyzed. For the sampling frequency of 1250 Hz used in this study, this translates to a range of 0.8 ms to 80 ms, which is a reasonable range for mammalian conduction delays in the central nervous system [51–53].

A prerequisite for Wiener’s principle of causality is the optimization of self-predictability of the target process *Y*. This is guaranteed by reconstructing the state space of the observables according to Takens’ delay embedding theorem [54] instead of analyzing the single univariate observables of the source and target time series. The two parameters d_x_ and τ in Eq (1) are optimized by applying a local constant predictor:

$${\hat{x}}_{t+\Delta t}^{d_{x},\tau }=\frac{1}{\left|U_{\varepsilon }\left(x_{t}^{d_{x},\tau }\right)\right|}\sum_{\left(x_{\theta }^{d_{x}\tau }\in U_{\varepsilon }\left(x_{t}^{d_{x},\tau }\right)\right)}x_{\theta +\Delta t}^{d_{x},\tau },$$ (11)

according to Ragwitz and Kantz [55], where ${\hat{x}}_{t+\Delta t}$ indicates a prediction of the state **x**_**t**_ Δt time steps ahead, $\left|{U_{\varepsilon }(x}_{t}^{d_{x}})\right|$ is the number of state vectors within the local spherical neighborhood U_ε_ of **x**_**t**_ with a diameter ε and **x**_ϴ_ represents the past states of **x**_**t**_ in U_ε_. In order to determine a suitable neighborhood one can either fix the diameter ε or the number of neighbors within U_ε_. For this study we fixed the number of neighbors to the TRENTOOL’s default value 4. This value has been suggested by [56] to be a good tradeoff between possible statistical and systematic errors when estimating TE. The dimension d_x_ and time delay τ were scanned over a range of 1 to 9 and 0.1 to 1 times the autocorrelation decay times (ACTs), respectively. The ACT is defined as the number of samples at which the autocorrelation function drops to 1/e [48]. The combination of d and τ that minimizes the root mean squared prediction error for one time step

$$RMSPE=\sqrt{\frac{\sum_{t=1}^{T}{\left({\hat{x}}_{t+\Delta t}^{d_{x},\tau }-x_{t+\Delta t}^{d_{x},\tau }\right)}^{2}}{T}}$$ (12)

is chosen for further analysis.

In order to estimate the individual probabilities given on the right hand side of Eq (10), TRENTOOL applies the nearest neighbor technique of Kraskov, Stögbauer and Grassberger [57]. In general, nearest neighbor techniques quantify the number of nearest neighbors of every point in a d-dimensional space, given a predefined neighborhood diameter ε. As different dimensional spaces are involved in TE estimation (d_x_ and d_y_ in Eq (10)), the Kraskov-Stögbauer-Grassberger algorithm corrects for the arising bias by fixing the number of neighbors for the highest dimensional space, i.e. the second term in Eq (10), and projects the resulting distances to all lower dimensional spaces. Incorporating the Kraskov-Stögbauer-Grassberger algorithm, the TRENTOOL estimator as given in Eq (8) can then be written as

$$TE_{SPO}\left(X\to Y\right)=\psi \left(k\right)+\langle \psi \left(n_{y_{t-1}^{d_{y}}}+1\right)-\psi \left(n_{y_{t}y_{t-1}^{d_{y}}}+1\right)-\psi \left(n_{y_{t-1}^{d_{y}}x_{t-u}^{d_{x}}}+1\right)\rangle ,$$ (13)

where ψ is the digamma function, k is the fixed number of neighbors in the highest dimensional space, n_ij_ is the number of neighbors in the spaces spanned by the subspaces i and j, and 〈.〉 denotes the average over time.

### Models

The generating models behind real electrophysiological recordings, especially for integrated quantities such as local field potentials (LFPs) from electroencephalography (EEG) or intracranial measurements, and the way how different neuronal populations encode and transmit information are usually not well understood [58]. To account for different types of electrophysiological data we applied a simulation framework using three different models: 1) the linear Kus-model [43], 2) a coupled logistic map model (CLMM) [44,59,60] and 3) a coupled sigmoid equations model (CSEM) [45]. The three different models are explained in detail in the following sections. We decided to use the linear Kus-model as it allows to use real EEG data as input and thus resembles large scale neural activity. The CLMM was chosen in order to specifically test the influence of filtering on TE estimation for highly nonlinear coupling dynamics. The CSEM was used to simulate small scale neural activity, i.e. single neuron activity. For both CLMM and CSEM we tested the influence of three different low-pass filters. The modeled delay δ was estimated from the simulated data by scanning over a range of u = 1–10 samples for the Kus-model and the CLMM. For the CSEM we also tested if the filter application might lead to a larger delay deviation so that we scanned a larger range of u = 1–100 samples.

For the remainder of this paper let X_i_ be the i^th^ channel of a system of coupled stochastic dynamic equations, γ = 0.4 the coupling factor, δ = [4,6,8] the delay of information transmission in samples, η a Gaussian white noise process with unit variance, V = 0.25*σ^2^ a pre-factor to scale η to one quarter of the variance σ^2^ of the first channel and f a coupling function. For every model and tested preprocessing technique we simulated 100 data sets each consisting of 20 trials.

#### Modified linear Kus-model

**Fig 1:**
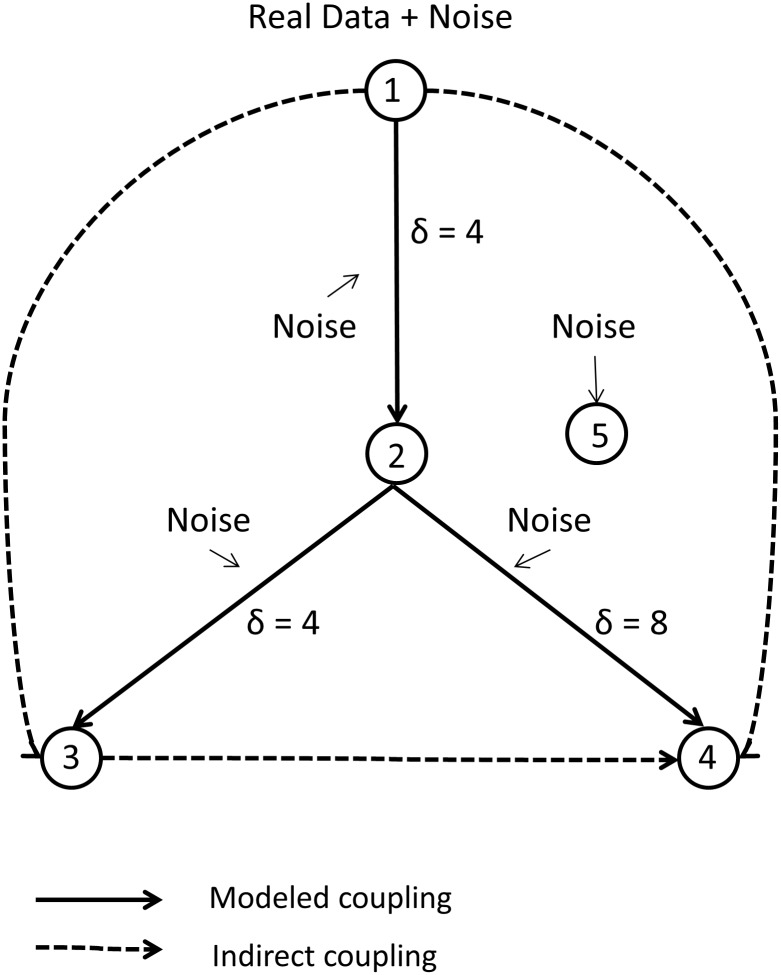
Coupling scheme for the modified Kus-model.

The original Kus-model is a network of six channels modeled by linearly coupled stochastic difference equations and one uncoupled channel consisting of Gaussian white noise [43]. The advantage of this model is its overall simplicity on the one hand and the possibility to implement real electrophysiological data on the other hand. In order to compare the different models, we used a similar structure for all models and in order to limit computational time we restricted the simulation to four channels plus one noise channel. Similar to the study of Florin et al. [36] for Granger causality, the original delays were altered from unit lag to 4 and 8 lags to test for the influence of different downsampling factors, and additionally to test for the effect of filtering on the TE estimation of interaction delays. The final model was hierarchically organized as shown in Fig 1

2.4s of EEG data plus Gaussian white noise is used as input (channel 1). The data is then time-shifted by the interaction delay of δ = 4 samples and independent white noise is added to generate channel 2 and so forth for channels 3 and 4. Channel 5 is independent white noise. External white noise (not shown) is added to all channels. Direct and indirect couplings are indicated by solid and dashed arrows, respectively. with the set of equations

$$\begin{array}{l} {\text{X}}_{1}\left(t\right)=d\left(t\right)+V\eta_{1}\left(t\right)\\ {\text{X}}_{2}\left(t\right)=0.4{\text{X}}_{1}\left(t-4\right)+V\eta_{2}\left(t\right)\\ {\text{X}}_{3}\left(t\right)=0.4{\text{X}}_{2}\left(t-4\right)+V\eta_{3}\left(t\right)\\ {\text{X}}_{4}\left(t\right)=0.4{\text{X}}_{2}\left(t-8\right)+V\eta_{4}\left(t\right)\\ {\text{X}}_{5}\left(t\right)=V\eta_{5}\left(t\right). \end{array}$$ (14)

For the input to the first channel, 2.4 seconds (≙ 3000 samples) of a previously recorded single channel EEG recording (d) of a Parkinson’s patient, sampled at a frequency of 1250 Hz were used. At every time step t, independent Gaussian white noise η_i_ scaled to one quarter of the variance of d was added to each channel to simulate internal noise. External noise was simulated by adding Gaussian white noise with zero mean scaled to one quarter of the variance of d to every channel. In contrast to the external noise, the internal noise becomes part of the dynamics of each connected channel. For each trial we used the same EEG data set as input but new internal and external noise was generated. The overall structure of the model allows for the generation of an arbitrary number of independent realizations of stochastic processes resembling electrophysiological data. Also, the combination of only one EEG channel with white noise, in contrast of using several EEG channels, controls for unknown causality structures in the model. Thus, only the predefined information flow is present in the network.

#### CLMM

**Fig 2:**
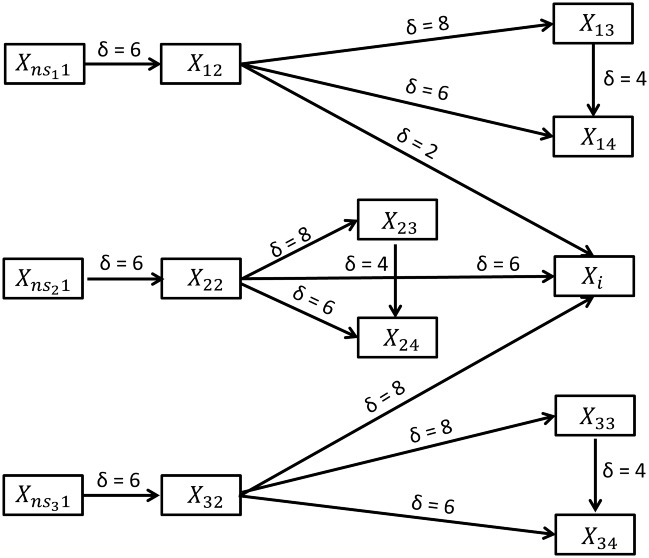
Coupling scheme for the CLMM. The first index of each channel denotes the number of times the logistic map is iterated, while the second index denotes the channel index within each of the three resulting branches. As X_i_ receives input from X_12_, X_22_ and X_32_ it is considered to be part of all three branches. Gaussian white noise ns is used as input (channel X_ns1_). The data is then time-shifted by the interaction delay of δ = 6 samples, passed to the logistic coupling function and independent white noise is added to generate channel X_*2_ and so forth. External white noise (not shown) is added to all channels.

The purpose of the CLMM was to explore the influence of filtering on TE estimation using a network of nodes with different degrees of nonlinear coupling. The CLMM consisted of uni-directionally coupled stochastic difference equations. The coupling scheme for the model is shown in Fig 2.

The input to the model was Gaussian white noise normalized to an interval between zero and one. The 13 channels were coupled by the logistic map [44]

$$f:{\text{x}}_{t,n+1}=\alpha {\text{x}}_{t,n}\left(1-{\text{x}}_{t,n}\right),$$ (15)

where i denotes the channel, n the number of subsequent iterations of the logistic map and t the time. The parameter α was set to 3.576, which corresponds to weak chaotic behavior.

**Fig 3:**
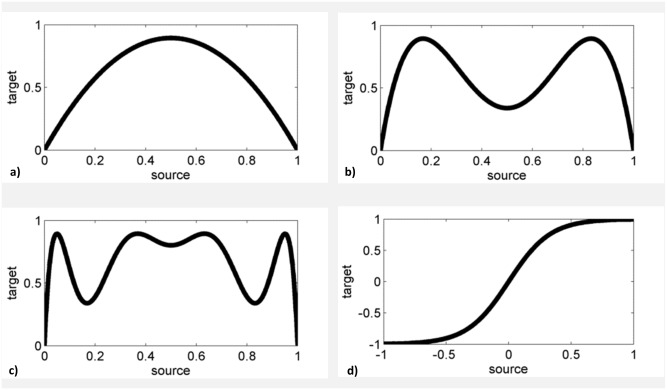
Coupling functions for the nonlinear models. a) Coupling function for the CLMM and n = 1 iteration. b) Coupling function for the CLMM and n = 2 iterations. c) Coupling function for the CLMM and n = 3 iterations. d) Coupling function for the CSEM.

The logistic map was used because of its simplicity and well understood parameter dependent non-divergent long time behavior. It is commonly used in simulation frameworks as a model of choice (see [61,62] for examples in the context of TE and [59,60] for simulation of neuronal activity). Our model was composed of three main branches with four channels each and an additional channel to which every branch projects with a different interaction delay. In order to simulate the drive of three complex signals with different interaction delays to a common output, the channel *X*_*i*_ receives input from each of the three branches and was thus considered to be part of every branch. Note that the three successive iteration steps resulted in the logistic coupling function to be of order two, four and eight. Fig 3a–3c shows the graphs of the CLMM’s coupling function for the three iteration steps.

Mathematically the channels were coupled according to the following equations:

$$\begin{array}{l} {\text{X}}_{ns1}\left(t\right)=\eta_{1}\left(t\right)\\ {\text{X}}_{m2}\left(t\right)=\frac{0.4f^{m}\left({\text{X}}_{m1}\left(t-6\right)\right)+V\eta_{2}\left(t\right)}{\gamma +V}\\ {\text{X}}_{m3}\left(t\right)=\frac{0.4f^{m}\left({\text{X}}_{m2}\left(t-8\right)\right)+V\eta_{3}\left(t\right)}{\gamma +V}\\ {\text{X}}_{m4}\left(t\right)=\frac{0.4\left(f^{m}\left({\text{X}}_{m2}\left(t-6\right)\right)+f^{m}\left({\text{X}}_{m3}\left(t-4\right)\right)\right)+V\eta_{4}\left(t\right)}{2\gamma +V}\\ {\text{X}}_{i}\left(t\right)=\frac{0.4\left(f^{1}\left({\text{X}}_{12}\left(t-2\right)\right)+f^{2}\left({\text{X}}_{22}\left(t-6\right)\right)+f^{3}\left({\text{X}}_{32}\left(t-8\right)\right)\right)+V\eta_{5}\left(t\right)}{3\gamma +V}, \end{array}$$ (16)

where f^m^ denotes m = 1 iterations of the logistic map for the first branch, m = 2 iterations for the second branch and m = 3 iterations for the third branch of the model.

Similar to the Kus-model internal Gaussian white noise was added as well as external Gaussian white noise both scaled to one quarter of the variance of the input signal. As the logistic map is only defined for an interval of 0 to 1, the internal as well as the external noise distribution were normalized to fit the interval. For the same reason X was rescaled at every time step t by dividing by the largest possible value namely ςγ+V with ς being the number of inputs.

#### Coupled sigmoid equations model (CSEM)

**Fig 4:**
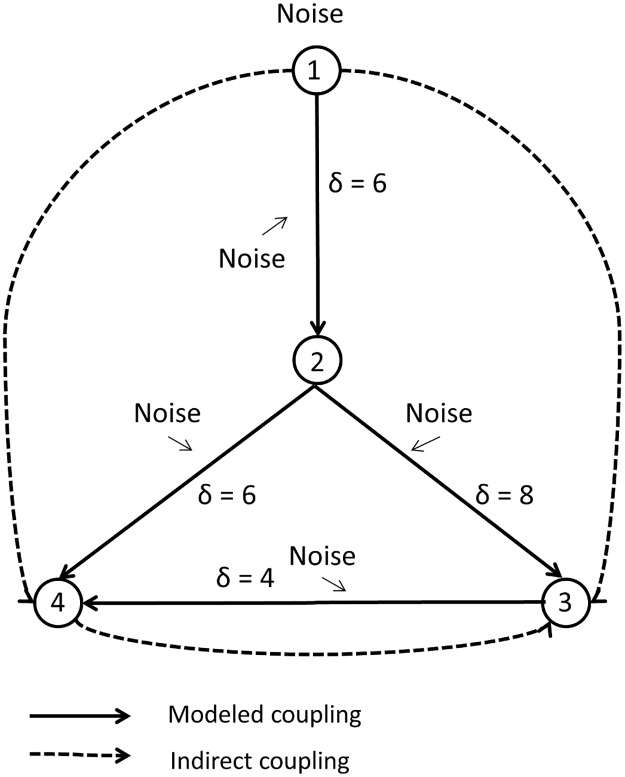
Coupling scheme for the CSEM. Gaussian white noise is used as input (channel 1). The data is then time-shifted by the interaction delay of δ = 6 samples, passed to the sigmoid coupling function and independent white noise is added to generate channel 2 and so forth for channels 3 and 4. External white noise (not shown) is added to all channels. Direct and indirect couplings are indicated by solid and dotted arrows, respectively.

The CSEM consisted of five uni-directionally coupled channels. Similar to the Kus-model the target channels were coupled by a linear combination of the past values of the source channels. All inputs to a channel were summed up, scaled by a prefactor of 0.4 similar to the other models and passed to a sigmoid function

$$f:y_{t}=\frac{2}{1+e^{-a{\text{x}}_{t}}}-1,$$ (17)

with parameter a = 6, which controlled the steepness of the sigmoid. The minimum and maximum of the sigmoid function was -1 and 1, respectively (Fig 3d). This model was chosen because of its biological importance, as the sigmoid function’s inflection point is a crude simulation of a neuron’s firing threshold. For the same reason, it is often employed in artificial neural networks [45]. For this model the interaction delay was scanned over a much broader range in order to test the influence of filtering on the delay deviation. We tested a range of u = 1–100 lags, which translates to a delay interval of 0.8 ms to 80 ms. The overall structure of the model is similar to one branch of the CLMM and is depicted in Fig 4.

The model is mathematically described as

$$\begin{array}{l} {\text{X}}_{1}\left(t\right)=\eta_{1}\left(t\right)\\ {\text{X}}_{2}\left(t\right)=f\left(0.4{\text{X}}_{1}\left(t-6\right)\right)+V\eta_{2}\left(t\right)\\ {\text{X}}_{3}\left(t\right)=f\left(0.4{\text{X}}_{2}\left(t-8\right)\right)+V\eta_{3}\left(t\right)\\ {\text{X}}_{4}\left(t\right)=f\left(0.4({\text{X}}_{2}\left(t-6\right)+{\text{X}}_{3}\left(t-4\right)\right))+V\eta_{4}\left(t\right). \end{array}$$ (18)

Gaussian white noise with zero mean was used as input to the system. Internal and external white noise with zero mean and variance scaled to one quarter of the input signal’s variance was added at each time step t and after calculation of the whole model, respectively.

### Preprocessing

The main goals of this study were to analyze the influence of filtering and downsampling on the estimation of TE. For this purpose, three different low-pass filter cut-offs at 320 Hz, 160 Hz, and 80 Hz and two high-pass filter cut-offs at 1 and 2 Hz were tested. For these tests, non-phase neutral also known as causal infinite impulse response (IIR) Butterworth filters of order 4 were used. We used Butterworth filters as they are the most common filters applied in neuroscience. They are characterized by a maximally flat frequency response in the pass band and a monotonic roll off into the stop band, i.e. no ripples in either pass- or stop band [63].

Additionally, three downsampling tests with factors of two, six, and ten were carried out. In order to have consistent trial lengths of 3000 samples per channel appropriately longer data sets were simulated before downsampling, i.e. trial lengths of 6000, 18000 and 30000 samples per channel, respectively. While, in a realistic setting, decimation would naturally lead to a reduction of data points, we were especially interested in the effects of subsampling and anti-aliasing filters. Thus, in order to isolate these effects, we kept data length constant. The finite sample effects on transfer entropy estimation have been previously described in [64,65]. The filter and downsampling was implemented using the *filter* and *decimate* functions of the Signal Processing Toolbox (v. 6.22) in Matlab (Matlab 2014b). Note that the decimate function also includes an IIR Chebyshev I filter of order 8 with a cut-off frequency of f_c_ = 0.8f_ny_/r, where r is the downsampling factor and f_ny_ the Nyquist frequency, as an anti-aliasing filter. This low-pass filter is applied in both forward and backward direction to the data so that it causes no phase distortion. Therefore, it is a non-causal filter application.

As secondary objective we wanted to compare phase neutral, i.e. non-causal, to non-phase neutral, i.e. causal, filtering procedures. For this comparison, we additionally used phase neutral filtering and tested three groups of low-pass filtered data with cut-off frequencies of 320 Hz, 160 Hz and 80 Hz and two groups of high-pass filtered data with cut-off frequencies of 1 and 2 Hz. For this comparison the Kus-model was used. We applied phase neutral filtering using the *filtfilt* function in Matlab, which applies the filter forward and backwards on the signal, thus restoring any phase distortions of the non-phase neutral Butterworth filter. As phase neutral filtering using *filtfilt* temporally aggregates twice as much samples as the *filter* function and thus effectively doubles the order of the filter, filters for phase neutral filtering procedures were generated as second order filters to match the order of the non-phase neutral filters. Finally, we hypothesized, that the filter order may have a significant impact on delay estimation, as with increasing filter orders more adjacent time points get aggregated. To test this, 80 Hz low-pass filtered datasets with increasing filter orders from 1 to 9 were compared to an unpreprocessed control dataset. All filters were IIR Butterworth filters implemented with the Signal Processing Toolbox in Matlab. The analyzed preprocessing types are summarized in Table 1.

**Table 1:** Analyzed preprocessing types.

Feature	Filter-Type	Hz	Filter-Order	Models
Control				Kus, CLMM, CSEM
Downsampling + Low-Pass (non-causal)	Chebyshev I	Fs: 125	8	Kus, CLMM, CSEM
		Ff: 60		
		Fs: 208		Kus, CLMM, CSEM
		Ff: 100		
		Fs: 625		Kus, CLMM, CSEM
		Ff: 300		
High-Pass (causal)	Butterworth	1	4	Kus, CLMM, CSEM
		2		Kus, CLMM, CSEM
Low-Pass (causal)		320		Kus, CLMM, CSEM
		160		Kus, CLMM, CSEM
		80		Kus, CLMM, CSEM
		80	1–9	Kus
High-Pass (non-causal)		1	2	Kus
		2		
Low-Pass (non-causal)		320		Kus
		160		Kus
		80		Kus

Fs: sampling frequency, Ff: filter cut-off frequency.

### Statistics

For each individual data set the estimated couplings were tested for statistical significance by applying a nonparametric permutation test using trial randomized surrogates (for details see [48]): First TE was calculated for each trial of each original dataset. Then surrogates were generated by shuffling the target time series between trials. TE was estimated for each trial of surrogate data and the mean difference between TE of the original data and surrogate data was calculated. Finally, TE results of each trial were randomly swapped between the original data and the surrogate data and the mean difference is calculated again. If the difference of mean TE between original and surrogate data fell above the 95 percentile of the distribution of the differences of permuted results, TE was considered significant at an alpha level of 5%. Note that, while theoretically TE should always be positive or zero, due to an estimation bias, estimated values of TE might be slightly negative if true TE values are close to zero [56]. This is, however, not a problem as long as the estimated TE values are significantly larger than the surrogate TE values.

The number of permutations limits the minimum possible p-value that can be calculated according to

$$\frac{1}{number\;of\;permutations}<\frac{p}{c},$$ (19)

where p is the minimum requested probability corrected for the number of statistical comparisons c. For this study we used TRENTOOL’s default number of permutations, i.e. 190100.

For the comparison of false detections, i.e. false negative and false positive connections, between preprocessing techniques and over all data sets Fisher’s exact test was applied, as this test is generally valid even for small sample sizes. The significance level of 5% was adjusted using Bonferroni correction to accommodate for multiple testing. The comparison of mean delay deviations between groups was performed using the nonparametric Wilcoxon rank-sum test with a subsequent Bonferroni correction at a significance level of 5%. A nonparametric test was chosen as normality could not be verified using the Kolmogorov-Smirnov test. For these tests the Statistics and Machine Learning Toolbox (v. 10.0) implemented in Matlab (Matlab 2014b) was used.

For every model four parameters were extracted from the data:

1. false negative direct connections (FNDC), which are presented as the percentage of total direct connections simulated per group.
2. false negative indirect connections (FNIC), which were calculated from estimated positive indirect connections p_ind,est_ and expected positive indirect connections p_ind,exp_ according to
  $$FNIC=100\left(1-\frac{p_{ind,est}}{p_{ind,exp}}\right).$$ (20)
3. false positive connections (FP), as percentage of the total amount of possible false positive connections.
4. mean deviation dd from the modeled interaction delay
  $$dd=\frac{1}{nm}\sum_{i=1}^{n}\sum_{j=1}^{m}\left|\delta_{est}^{ij}-\delta_{exp}^{j}\right|,$$ (21)
  where *n* indicates the number of generated data sets, *m* the number of simulated direct connections, $\delta_{est}^{ij}$ the estimated delay and $\delta_{exp}^{j}$ the expected modeled delay for each connection and data set. A mean delay deviation of zero indicates a perfect estimate of the true interaction delay.

## Results

### The Kus-model

**Fig 5:**
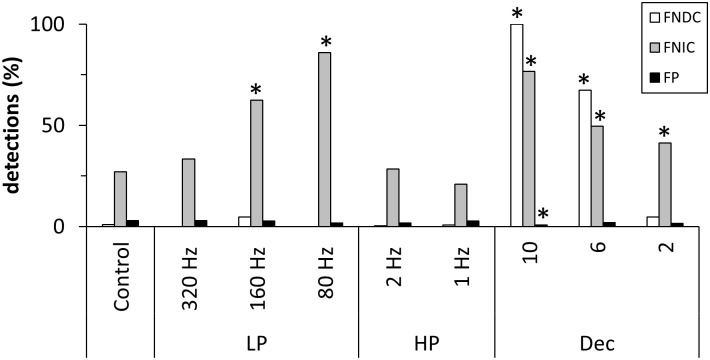
False detections in percent for the Kus-model. LP: low-pass filter, HP: high-pass filter, Dec: decimation. Asterisks indicate results significantly different from control (Fisher’s exact test, p < = 0.05, Bonferroni corrected).

In Fig 5 the influence of filtering and downsampling on the false detection rate using the Kus-model is presented. For the control and all filtered data sets FNDC and FP were detected to be below five percent. However, for FNIC filtering with progressively lower low-pass cut-off frequencies led to a significant monotonic increase from 27% for the control group up to 86% for the 80 Hz low-pass filter.

Downsampling had the greatest influence on false detections. A sampling factor of ten resulted in a significant increase of 100% FNDC and nearly 80% FNIC. A sampling factor of six resulted in a similar but slightly smaller significant increase of FNDC and FNIC with 67% and 50%, respectively. In contrast, a downsampling factor of 2 only led to a significant increase of FNIC to 41%.

**Fig 6:**
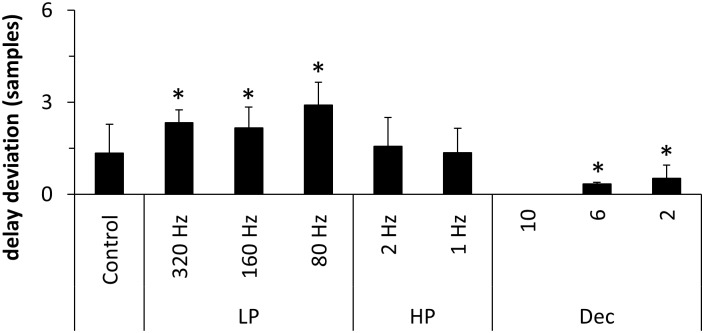
Delay deviation in samples for the Kus-model. LP: low-pass filter, HP: high-pass filter, Dec: decimation. Asterisks indicate results significantly different from control (Wilcoxon rank-sum test, p < = 0.05, Bonferroni corrected). Error bars indicate standard deviation (SD).

In Fig 6 the same influences were analyzed with respect to the estimation of the modeled interaction delay. For the control group and both high-pass filter groups the mean delay deviation was below 2.0 samples. Concerning the effect of low-pass filtering on the estimation of the mean delay deviation, our results could not demonstrate a clear monotonic increase with decreasing low-pass filter cut-off frequency as one might have expected. However, for all low-pass filtered groups the delay deviation was significantly larger than for the control group with the maximum value of 2.9 [±0.7] samples for the 80 Hz low-pass filter. Downsampling using factors six and two led to a significant decrease of delay deviation. This is because the observed deviation from the modeled delay generally led to an underestimated delay. However, since downsampling leads to a decrease of the modeled interaction delay in terms of samples the range of possible deviations also decreases. As no modeled connections could be detected after downsampling with a factor of 10, no delay deviation could be analyzed for this group.

**Fig 7:**
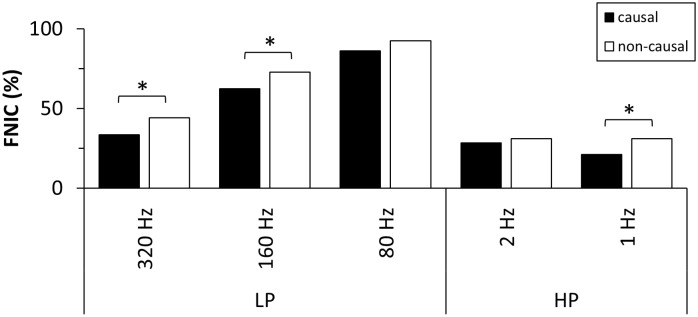
Comparison of FNIC for causal and non-causal filtering procedures using Butterworth filters. LP: low-pass, HP: high-pass. Asterisks indicate significant differences (Fisher’s exact test, p < = 0.05, Bonferroni corrected).

We also tested the influence of filtering using phase neutral, i.e. non-causal, filters on TE estimation. In Fig 7 causal and non-causal filtering are compared with respect to detected FNIC. Both filtering procedures showed a monotonic increase in FNIC with decreasing cut-off frequency. In comparison to causal filtering significantly more FNIC were detected for non-causal low-pass filtering at 320 Hz and 160 Hz and the non-causal high-pass filtering at 1 Hz. FNDC and FP were below 5% for each cut-off frequency and showed no significant differences (S4 and S5 Figs).

**Fig 8:**
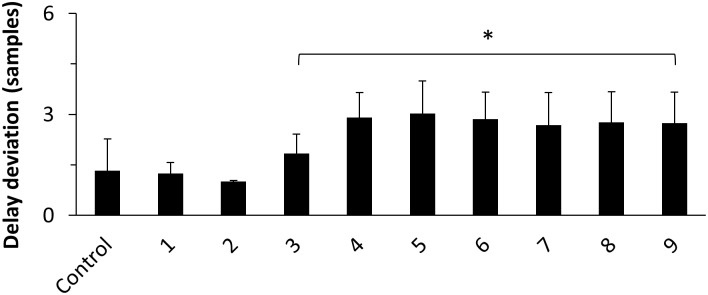
Delay deviation in samples for the Kus-model and increasing low-pass filter orders. Asterisks indicate results significantly different from control (Wilcoxon rank-sum test, p < = 0.05, Bonferroni corrected). Error bars indicate standard deviation.

Finally, we hypothesized that the filter order may have a significant influence on delay estimation as with higher orders more adjacent time points get temporally aggregated. To test this, we compared nine 80 Hz low-pass filtered datasets with increasing filter orders from 1 to 9 with an unpreprocessed control group. The results are summarized in Fig 8. Filter orders from 3 to 9 led to a significant increase of delay deviation in comparison to control. Filtering always led to an underestimation of modeled interaction delay. Note that for all filter orders FNDC and FP were below 4% (S1 and S3 Figs). FNIC ranged from 50% for filter order 1 to 93% for order 9 (S2 Fig).

### The CLMM

**Fig 9:**
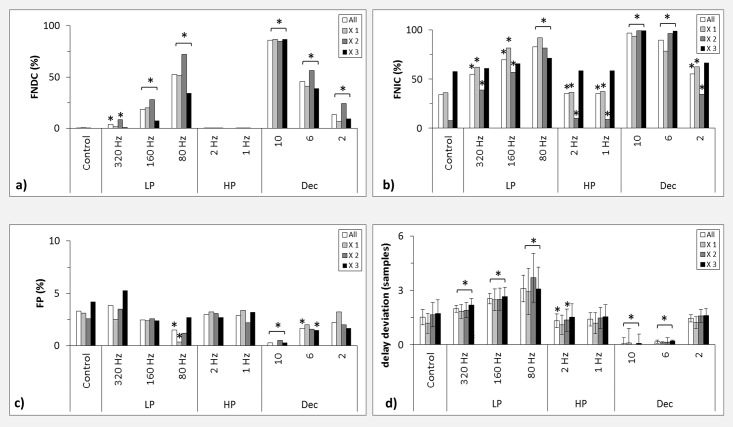
Results for the CLMM. False detections in percent for different branches of CLMM reflecting different degrees of nonlinearity for a) FNDC, b) FNIC, and c) FP. d) Delay deviation in samples for different degrees of nonlinearity. LP: low-pass filter, HP: high-pass filter, Dec: decimation, X i: ith branch of the CLMM, All: average results for all degrees of nonlinearity. Asterisks indicate results significantly different from control (a-c: Fisher’s exact test, p < = 0.05, Bonferroni corrected, d: Wilcoxon rank-sum test, p < = 0.05). Error bars indicate standard deviation.

In Fig 9 the influence of filtering and downsampling on false detections as well as on the estimation of the interaction delay is shown for the different branches of the CLMM, which reflect different degrees of nonlinearity. For the FNDC (Fig 9a), a monotonic increase was observed for progressively lower cut-off frequencies and progressively larger downsampling factors. This behavior was independent of the degree of nonlinearity, i.e. the number of times the logistic map was iterated. Note, however, that the increase of FNDC was much smaller for the third and most nonlinear branch (34%) than for the second (72%) and first branch (52%). As Table 2 demonstrates, no single connection was responsible for this effect. However, if we compare branch X 3 only with X 1 then the strongest difference was observed for the connections X_m2_ to X_i_. (94% FNDC for X 1 and 28% for X 3), notably the only connection with different modeled interaction delay for all branches. For the control and high-pass filters FNDC were always below one percent (Fig 9a). Applying low-pass filters of 320 Hz, 160 Hz and 80 Hz resulted in FNDC of up to 9%, 28%, and 72%, respectively, for the second branch. Interestingly, it was the second and not the third branch that exhibited the overall highest percentages of filter dependent FNDC. Downsampling with factors 2, 6 and 8 led to significant increases of FNDC up to 24%, 57% and 87% respectively.

**Table 2:** Percent of significant FNDC per branch for the 80 Hz low-pass filter.

Branch	X 1	X 2	X 3
**X**_**ns1**_ **--> X**_**m2**_	82%	60%	93%
**X**_**m2**_ **--> X**_**m3**_	0%	37%	0%
**X**_**m2**_ **--> X**_**m4**_	8%	71%	4%
**X**_**m2**_ **--> X**_**i**_	94%	93%	28%
**X**_**m3**_ **--> X**_**m4**_	74%	100%	48%

For the FNIC (Fig 9b) a similar monotonic frequency dependent increase of detections was observed as for the FNDC. Also, FNIC did not increase with increasing degree of nonlinearity. However, even for the control group up to 58% FNIC were detected in the third branch of the model. In the first branch low-pass filters of 320 Hz, 160 Hz and 80 Hz resulted in up to 62%, 81% and 92% FNIC, respectively. Notably, in comparison to branch 1 and 2 the difference between low-pass filtering groups was much smaller in branch three. With the exception of branch 3 downsampling always led to a significant increase of FNIC in comparison to control. In branch 3 only factors 10 and 6 resulted in a significant increase of FNIC. High-pass filtering did not influence the occurrence of FNIC.

Fig 9c shows the percentage of FP for the different filter groups and different degrees of nonlinearity. Independent of the applied filter or the degree of nonlinearity, the FP were distributed around 2.5%, with the highest percentage of 5.3% for the third branch filtered with a 320 Hz low-pass and the lowest of 0.4% for the first branch filtered with an 80 Hz low-pass filter. Interestingly, after an initial rise of FP when applying a low-pass filter of 320 Hz, a monotonic decrease was observed when applying a 160 Hz or 80 Hz low-pass filter. This decrease was observed for all branches, i.e. it was independent of the degree of nonlinearity. However, this decrease was only significant for the 80 Hz low-pass filter and only for the averaged results and the first branch of the model. A similar behavior was observed for increasing downsampling factors, where we observed a monotonic decrease of FP. For averaged results and all branches we observed a significant decrease of FP for a downsampling factor of ten and also for a factor of 6 in the third branch.

A significant monotonic increase of the mean delay deviation ranging from 1.2 [± 0.6] up to 3.7 [± 1.4] samples was observed after application of low-pass filters with gradually lower cut-off frequency (Fig 9d). Larger downsampling factors resulted in a significant monotonic decrease of delay deviation ranging from 1.2 [±0.3] for a factor of 2 to 0.0 [±0.0] for a factor of 10. This behavior was independent of the degree of modeled nonlinear coupling.

### The CSEM

**Fig 10:**
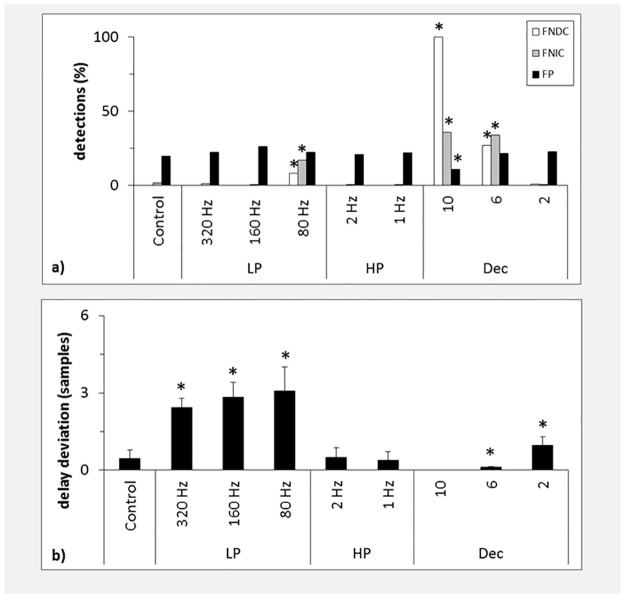
Results for the CSEM. a) False detections in percent for the CSEM. b) Delay deviations for the CSEM. LP: low-pass filter, HP: high-pass filter, Dec: decimation. Asterisks indicate results significantly different from control (a: Fisher’s exact test, p < = 0.05, Bonferroni corrected, b: Wilcoxon rank-sum test, p < = 0.05). Error bars indicate standard deviation.

Fig 10a displays the percentage of false detections for the CSEM. Of the applied filters only the 80 Hz low-pass filter resulted in a significant increase of FNDC and FNIC, with 8% and 17%, respectively. For all groups including the control up to 26% FP were detected. In accordance with the Kus and CLMM downsampling led to a significant increase of FNDC and FNIC for downsampling factors of 10 and 6. However, no increase was detected for a factor of 2. Interestingly, downsampling with a factor of 10 led to much fewer FNIC than FNDC (36% and 100%, resp.). A similar trend could be observed for the Kus-model. Again, downsampling resulted in a monotonic decrease of FP for increasing downsampling factors. However, this decrease was only significant for a factor of 10.

**Fig 11:**
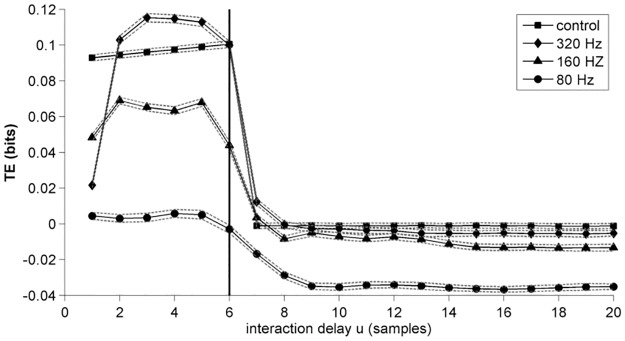
Influence of filtering on delay estimation. Mean TE as a function of tested interaction delays for the coupling of channels 1 and 2 of the CSEM after low-pass filtering and for the control. Dotted lines represent standard deviation. The black bar at u = 6 indicates the modeled interaction delay. Note that, while TE is defined to be positive semidefinite, estimated TE can be negative due to estimation bias [57].

For the CSEM we estimated the interaction delays over a larger range of u = 1–100 lags in order to test whether delay deviations stay confined to the temporal vicinity of the true interaction delay or spuriously high deviations appear. Application of a 320 Hz low-pass filter led to a significant increase of delay deviations from 0.5 [±0.3] samples to 2.4 [±0.4] samples (Fig 10b). Low-pass filter of 160 and 80 Hz both led to an even stronger delay deviation of 2.8 [±0.6] and 3.1 [±0.9] samples, respectively. Concerning the high-pass filters, no differences could be detected in comparison to control. We observed an increase of delay deviation to 1.0 [±0.3] for a downsampling factor of 2 and a significant decrease to 0.1 [±0.0] for the factor 6. As no modeled connections could be detected for the factor 10, no delay deviations were tested. Fig 11 shows the mean TE as a function of tested interaction delays for the control and the 320 Hz, 160 Hz and 80 Hz low-pass filtered datasets. No spurious side peaks for delays larger than the modeled delay u = 6 were observed (delays u = 20–100 not shown). Note how filtering with successive lower cut-off frequencies leads to a decrease of estimated TE. Additionally, note how filtering results in a smoother decline of TE for u>6. While TE is in theory defined to be positive semidefinite, negative values are observed here due to the estimation procedure. In summary no spuriously high delays were detected for this larger scanning range. Similar to the Kus and the CLMM if a delay deviation was present, the interaction delay was almost always underestimated.

## Discussion

In this study we tested the influence of different preprocessing techniques including different filters and downsampling factors on the estimation of TE. To this end, we used one linear and two nonlinear models in order to simulate different kinds of neural activity. The present study demonstrates that preprocessing can have a tremendous effect on inferring directed information flow using TE estimation. The results are discussed in detail in the following paragraphs.

### Effects of filtering

We demonstrated that the influence of filtering on TE estimation is highly dependent on the network’s coupling dynamics. For the case of linear coupling as implemented in the form of the Kus-model, neither low-pass nor high-pass filtering led to a significant increase of detected FNDC or FP. However, low-pass filtering resulted in a significant cut-off frequency dependent monotonic increase of FNIC. A possible explanation for the negative influence of low-pass filtering may be that the amount of information transmitted over several channels was thinned out with the addition of dynamic noise at every time step and channel. At a critical amount of transferred information in the original signal low-pass filtering eliminated much of the remaining information content, which resulted in an increased number of missed connections or equivalently in a decrease of sensitivity of TE estimation. Thus, while the estimation of direct connections was robust under filtering, low-pass filtering seems to mainly affect the detection of indirect connections for a linear system.

For the nonlinear CLMM and CSEM we could demonstrate a monotonic filter cut-off frequency dependent increase of false negatives also for direct connections. For a strong nonlinearly coupled system one might expect the joint probability distribution of past states of the driving signal and present values of the driven signal to be widely spread out, with discrete isolated peaks, as similar states of the driving signal may lead to vastly different values of the driven signal (Fig 3). Therefore, similar states of the driving signal would be less predictive for similar values of the driven signal. In contrast, for linearly coupled signals one would expect broad peaks in the joint probability distribution as similar states of the driving signal would lead to similar states of the driven signal through the linear coupling function. Thus, if precise knowledge of the driver’s and driven system’s states is blurred through temporal aggregation by a filter function a lot of the transferred information may remain undetected depending on the time scale of predictability of the nonlinear system.

Using the CLMM we tested whether the effect of filtering was enhanced for progressively stronger degrees of nonlinearity. However, no general qualitative differences were observed. For the strongest nonlinear coupling, i.e. the third branch, we found that the increase of FNDC after low-pass filtering was much smaller than for the weaker nonlinear couplings (35% for the third branch vs. 72% for the second branch and 52% for the first branch).

For the CSEM in all data sets including the control a large amount (~20%) of FP were observed. This is not surprising as the sigmoid function acts as an additional filter, which effectively eliminates all predictive information for values of the source time series smaller -1 and larger 1. Thus, the high number may be the result of a finite sample effect of this specific model. Taking Bonferroni correction into account, the detected false positives were marginally considered significant in contrast to true positives found (e.g. CSEM control 1→2: mean p-value = 0.00006 vs. CSEM control 2→1 mean p-value = 0.04907).

Another general source for spurious connections is the connectivity structure of the model (Fig 4). The triangular motif in the lower half of the model introduced the notion of self-feedback to the system. Part of the information node four receives from channel two is transmitted six samples later via channel three. Thus, if one only observes node four this dynamics would appear as self-feedback with a delay of six samples. As Hahs and Pethel [66] pointed out spurious connections can be detected if an anticipatory element is present in the system. Wibral et al. [50] suggested that this problem should be taken care of by calculating TE between the signal in question and its own past. In practice it would also be possible to use a multivariate TE estimator [67].

Using the Kus-model Florin et al. [36] tested the influence of filtering on the estimation of different Granger-causality based methods. Consistent with our results the authors reported an overall cut-off frequency dependent significant increase of false negatives, but also an increase of false positives when applying 80 Hz and 160 Hz low-pass filters in comparison to an unfiltered control data set. Note that in that study indirect connections were also defined as FP. In comparison to squared partial directed coherence (sPDC), which was stated to be the most robust Granger causality measure, we detected far less FNDC for TE (< 3%) using an 80 Hz low-pass Butterworth filter in a range of filter orders from 1 to 9 (S1 Fig). For sPDC Florin et al. observed a monotonic increase from 2% to 28% for filter orders from 2 to 8. For the same filter parameters Florin et al. also observed a monotonic increase of FP from 1% to 12%. Again, we could not detect a similar effect for TE. FP were observed to be rather constant for different filter orders and always below 3.5% (S3 Fig). Incorporating FNIC into FP we observed an initial drop from 11.2% at filter order 1 to 5.8% at filter order 2. For the remaining filter orders (3–9) FP remained constant at 3.8% [± 0.5] (S6 Fig). Overall, for linear interactions, TE seems to be more robust than sPDC when filters are applied.

**Fig 12:**
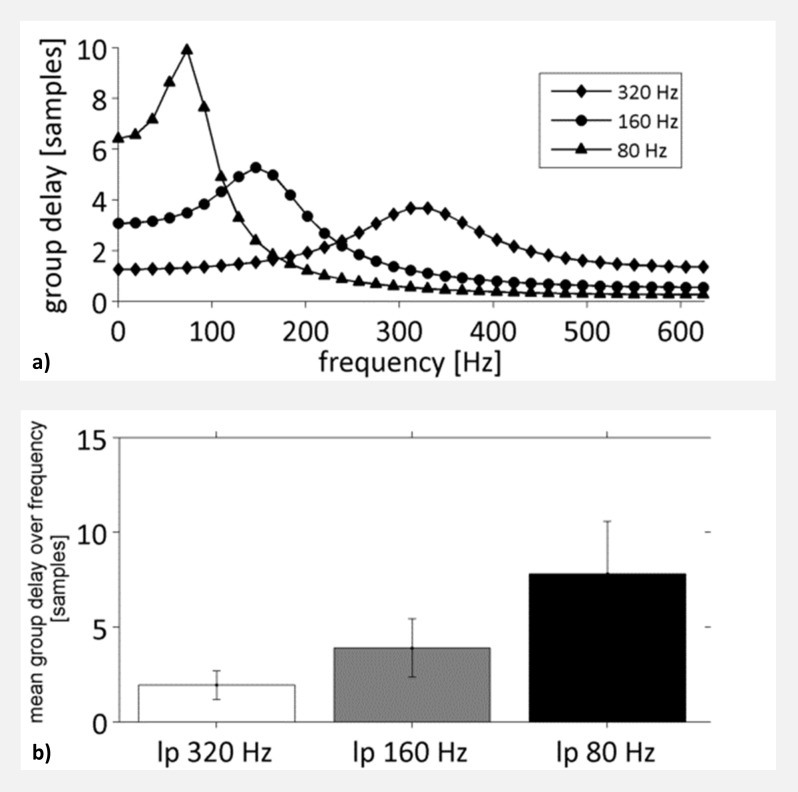
Group delay for different cut-off frequencies of a Butterworth low-pass filter. a) Group delay as a function of frequency for low-pass filter of 320 Hz (bottom), 160 Hz (middle) and 80 Hz (top). b) Mean group delay over pass-band frequency. lp: low-pass. Error-bars indicate standard deviation.

**Fig 13:**
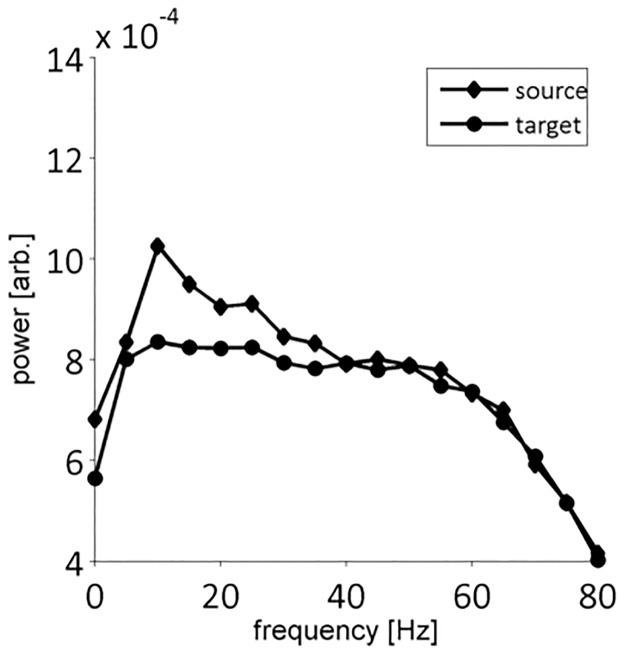
Mean power spectra of channel 2 (source) and channel 3 (target) low-pass filtered at 80 Hz. The mean is taken over 20 trials x 100 datasets.

Low-pass filtering had a significant influence on the estimation of the interaction delay. Generally, a filter dependent underestimation of interaction delay was observed in all channels and models. This delay deviation can be explained either by a forward shift of the target or a backward shift of the source time series. A reason for a forward shift might be the so called group delay of the applied filter, which is the negative first derivative of the filter’s phase response (Fig 12). However, if target and source activity get time shifted by the same factor no difference of interaction delay would be observed. This would be the case if source and target time series exhibit a highly similar power spectrum. If however the source time series shows higher power in distinct frequency bands than the target time series, the delay of these frequency bands would have a higher impact on overall transmission delay. As an example, (Fig 13) shows the mean power spectra of channel 2 (source) and channel 3 (target) of the Kus model low-pass filtered at 80 Hz. While the power spectrum of the target time series is flat in the range of 5–60 Hz, the source time series shows higher power in the range of 5–40 Hz with a distinct peak at 10 Hz. Accordingly, these observations might explain the detected transmission delay.

As the group delay is not only dependent on cut-off frequency but also on filter order we speculated that the filter order may be a significant factor of a filter’s influence on delay estimation, as with higher filter order more adjacent time points get temporally aggregated. We tested this by using an 80 Hz low-pass filter with filter orders from 1 to 9 and compared estimated interaction delays with an unfiltered control dataset. We found significant stronger delay deviations for filter orders between 3 and 9, although, after an initial increase of delay deviation at filter order 3, it stayed at approximately 2.8 samples for higher filter orders.

Not only the cut-off frequency is of practical importance, but also whether the filter is applied in a phase neutral manner, i.e. in forward and backward direction, or in a non-phase preserving manner, i.e. only in forward direction. It has been shown that phase neutral filters lead to a higher number of spurious or false connections when applying Granger based causality measures [36]. This may be because phase neutral filters temporally aggregate past and future time points, thus destroying the causality structure of the time series. In line with previous reports concerning Granger causality we could demonstrate that non-causal filtering leads to a higher number of false negative indirect connections when estimating TE. However, no significant differences could be detected concerning false negative direct connections or false positives.

From a computational point of view low-pass filtering allows for a distinct reduction of the phase-space dimensionality and thus significantly speeds up computation time. For the Kus-model filtering with a low-pass filter of 80 Hz led to a nearly six times shorter computation time. Even for the low-pass filter of 320 Hz computation time was accelerated by a factor of 3.4 (S7 Fig). However, as any filter has the potential to disturb inference on TE one should carefully consider if the accelerated computation time is worth taking the risk of detecting spurious causalities. Though computation time and the ‘curse of dimensionality’ are still highly relevant, progress in these issues is made continuously [68]. One recent approach is to use non-uniform embedding for phase-space reconstruction as it reduces redundancy by only incorporating past values of source and target time series into state vectors that are most predictive for the target’s present [69,70].

If one is interested in resolving information transfer in distinct frequency bands, one should refrain from filtering and consider using spectral measures of Granger causality as, so far, no spectral representation is possible for TE.

### Effect of downsampling

We demonstrated that low-pass filtering and successive downsampling with a sampling factor greater than the highest interaction delay can result in a very high percentage of FNDC. This is in line with the findings of Breitung and Swanson [71], Florin et al. [36] and Smirnov and Bezruchko [72], who got a similar result when analyzing the influence of downsampling on Granger-causality based methods.

Basically three possibilities can be thought of, how estimation of information transfer is disturbed. The first and most important possibility is the deletion of past states of the driver’s time series that are most informative for the prediction of the target’s time series present. If the downsampling factor covers the whole time span of an interaction delay, then no predictive information transfer can be estimated [73]. Even when the downsampling factor is smaller than the interaction delay, a significant amount of information may be lost depending on the information storage capabilities (see [74] for an introduction to information storage), i.e. the memory of the time series. If the target samples of the driven time series are cut out due to downsampling, information transfer might still be detected if the transferred information is stored long enough to be detected in the next available sample. The second possibility to disturb TE estimation is through the use of anti-aliasing low-pass filters. Although they are necessary in order to not violate the Nyquist criterion [46], which states that the sampling frequency should be at least twice as high as the highest expected frequency in the signal, low-pass filters can have the same negative influences on TE estimation as discussed in the previous paragraph. This may be the reason that for the CLMM even for the downsampling factor 2 a significant increase of FNDC was observed. Finally, downsampling results in a reduction of sample size. According to [64] and [65] finite sample effects may lead to the detection of spurious causalities. In this study however, sample size was kept constant for different downsampling factors which excludes finite sample effects as a possible reason for spurious detections. Generally speaking, we suspect that the effect of downsampling is different from filtering in the same way as for Granger causality, where it has been shown, that downsampling, but not causal filtering not only influences the estimation, but even distorts the population statistics of TE [37,38].

### Conclusion

Based on the results of this study, we recommend refraining from low-pass filtering and downsampling data when trying to infer directed information transfer by estimating TE. However, high-pass filtering does not influence TE estimation to a relevant point and can thus be used to preprocess data if slow drifts or movement artifacts are present. If filtering is necessary because of other known high frequency artifacts in the data or to speed up computation time, one has to keep in mind that causalities may remain undetected, depending on the filter’s cut-off frequency. Downsampling should only be done if knowledge about the system’s interaction delays exists and a sampling factor smaller than the longest interaction delay is chosen. While this study focused on the estimation of TE, future studies should try to answer the question if and how the population statistics of TE are influenced by filtering and downsampling.

## Supporting information

S1 Fig FNDC for the Kus-model as a function of filter order for an 80 Hz causal low-pass Butterworth filter. (TIFF)

S2 Fig FNIC for the Kus-model as a function of filter order for an 80 Hz causal low-pass Butterworth filter. (TIFF)

S3 Fig FP for the Kus-model as a function of filter order for an 80 Hz causal low-pass Butterworth filter. (TIFF)

S4 Fig Comparison of FNDC for causal and non-causal Butterworth filter using the Kus-model. (TIFF)

S5 Fig Comparison of FP for causal and non-causal Butterworth filter using the Kus-model. (TIFF)

S6 Fig FP for the Kus-model including detected indirect connection as a function of filter order for a causal 80 Hz low-pass Butterworth filter. (TIFF)

S7 Fig Computation time for 100 datasets of the Kus-model each with 20 trials for different Butterworth low-pass filter applications. Computation was done on two Dell precision tower 7910 XCTO base each with two Intel Xeon processors E5-2670 v3 (12 cores HT, 30 MB cache, 2.3 GHz) and 64 GB RAM.
(TIFF)
