# Supplementary figures and images for: The influence of filtering and downsampling on the estimation of transfer entropy
Source: PLoS One. 2017 Nov 17;12(11):e0188210. doi: 10.1371/journal.pone.0188210 (PMC5693301; doi:10.1371/journal.pone.0188210)

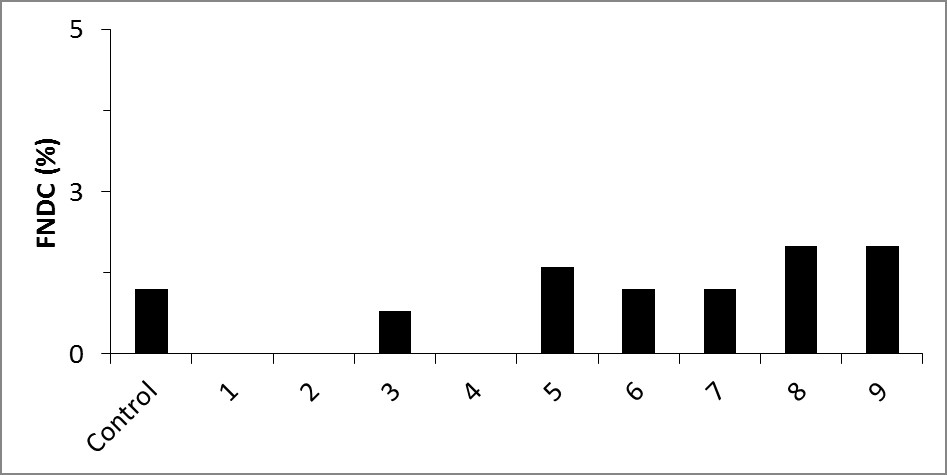

Supplement: S1 Fig — (TIFF) [file pone.0188210.s001.tiff]

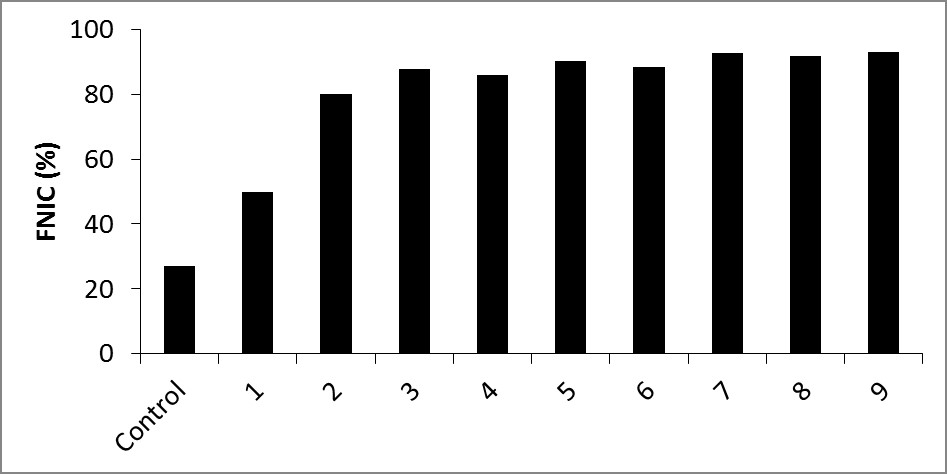

Supplement: S2 Fig — (TIFF) [file pone.0188210.s002.tiff]

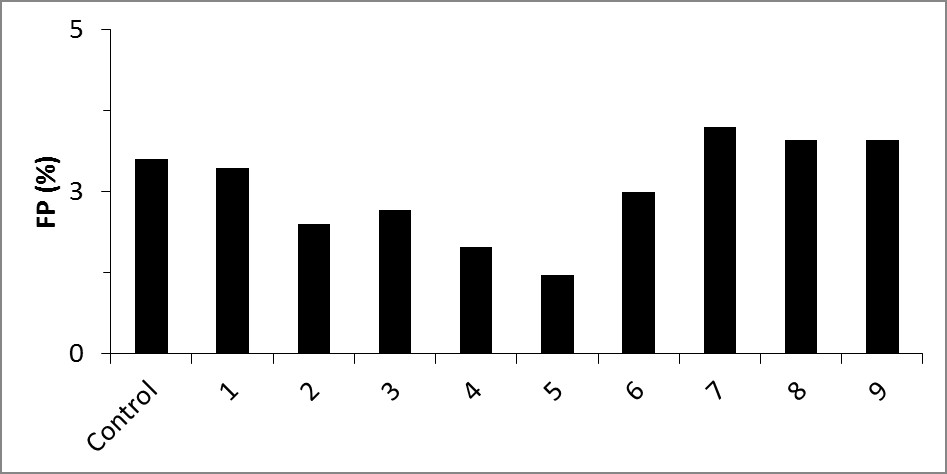

Supplement: S3 Fig — (TIFF) [file pone.0188210.s003.tiff]

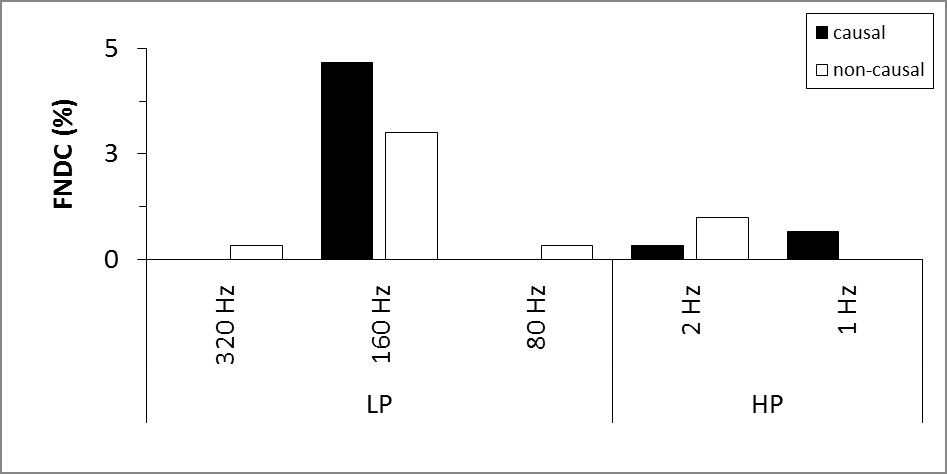

Supplement: S4 Fig — (TIFF) [file pone.0188210.s004.tiff]

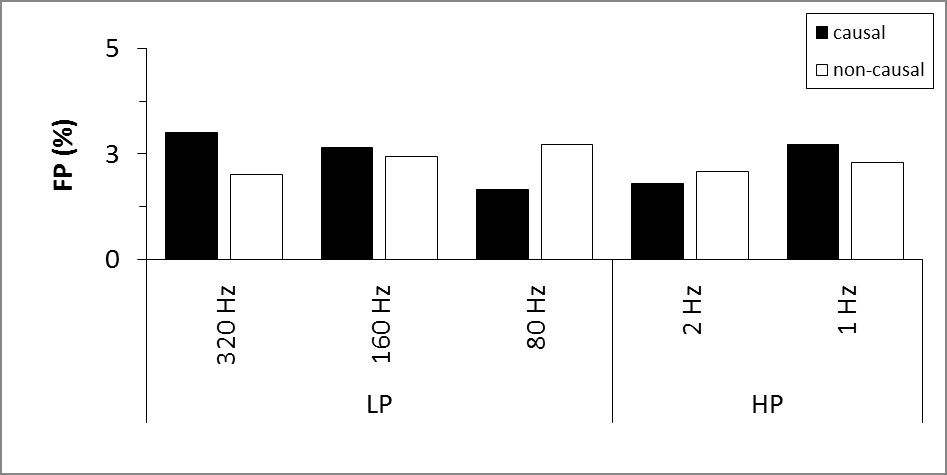

Supplement: S5 Fig — (TIFF) [file pone.0188210.s005.tiff]

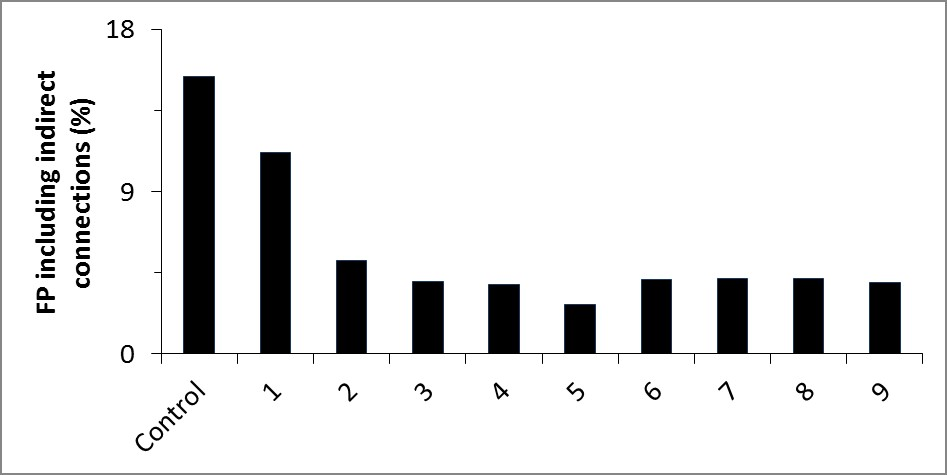

Supplement: S6 Fig — (TIFF) [file pone.0188210.s006.tiff]

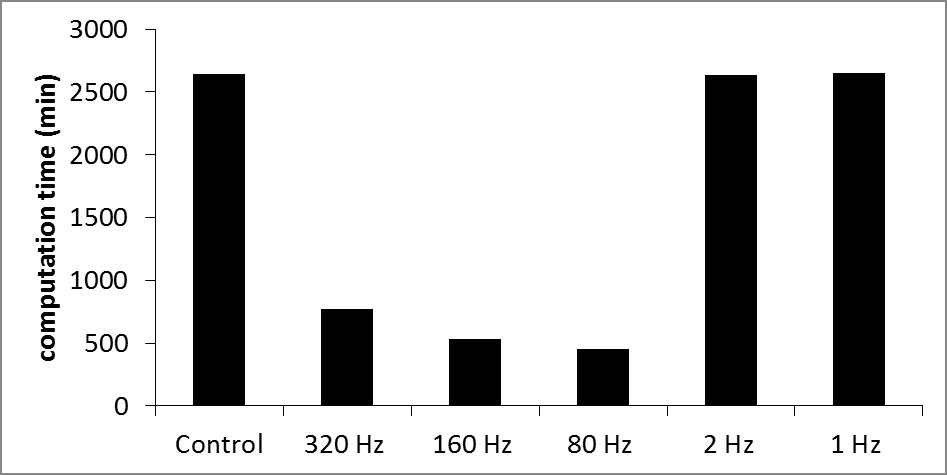

Supplement: S7 Fig — Computation was done on two Dell precision tower 7910 XCTO base each with two Intel Xeon processors E5-2670 v3 (12 cores HT, 30 MB cache, 2.3 GHz) and 64 GB RAM. (TIFF) [file pone.0188210.s007.tiff]
